# Premature ovarian aging in *BRCA* carriers: a prototype of systemic precocious aging?

**DOI:** 10.18632/oncotarget.24638

**Published:** 2018-03-23

**Authors:** Irit Ben-Aharon, Mattan Levi, David Margel, Rinat Yerushalmi, Shulamith Rizel, Shlomit Perry, Eran Sharon, Noa Hasky, Ronit Abir, Benny Fisch, Ana Tobar, Ruth Shalgi, Salomon Marcello Stemmer

**Affiliations:** ^1^ Institute of Oncology, Davidoff Center, Rabin Medical Center, Beilinson Campus, Petach Tikva, Israel; ^2^ Department of Cell and Developmental Biology, Sackler Faculty of Medicine, Tel-Aviv University, Ramat Aviv, Tel-Aviv, Israel; ^3^ Department of Surgery, Rabin Medical Center, Beilinson Campus, Petach Tikva, Israel; ^4^ IVF and Infertility Unit, Schneider Women Hospital, Rabin Medical Center, Beilinson Campus, Petach Tikva, Israel; ^5^ Sackler Faculty of Medicine, Tel-Aviv University, Ramat Aviv, Tel-Aviv, Israel; ^6^ Department of Pathology, Rabin Medical Center, Petach Tikva, Israel

**Keywords:** BRCA, ovarian aging, systemic precocious aging

## Abstract

**Purpose:**

Though former evidence implies a correlation of breast cancer susceptibility gene (*BRCA*) mutation with reduced ovarian reserve, the data is yet inconsistent. Our aim was to investigate biomarkers of ovarian aging in a cohort of young healthy carriers of the *BRCA* mutation. We hypothesized that the role played by *BRCA* genes in aging pathways is not exclusive to the ovary.

**Experimental Design:**

Healthy female *BRCA* carriers, 40 years or younger and healthy male *BRCA* carriers, 50 years or younger, were enrolled in the study. Serum anti-mullerian Hormone (AMH), fibroblast growth factor-23 (FGF-23), Klotho and IL-1 were measured by enzyme-linked immunosorbent assay (ELISA). Ovarian AMH and protein kinase B (AKT) mRNA from *BRCA* carriers who underwent prophylactic oophorectomy and from age-matched, healthy, non-carriers who underwent partial oophorectomy due to benign conditions were analyzed by qPCR.

**Results:**

Thirty-three female (median age 35y) and 20 male (44y) *BRCA* carriers were enrolled into the study and matched to control non-carriers (34y and 43y, respectively). Serum AMH level was significantly lower in *BRCA* female carriers than in both non-carrier controls and age-matched nomograms. The levels of ovarian AMH and AKT mRNA were significantly lower in carriers than in controls. The systemic aging cytokines FGF-23, klotho and IL-1 displayed a differential expression in carriers of both genders. FGF-23 level was higher in carriers (P=0.06).

**Conclusions:**

Our results suggest a link between *BRCA* mutation, accelerated ovarian aging and systemic aging-related pathophysiology.

## INTRODUCTION

Germline mutations in the two known breast cancer susceptibility genes; breast cancer susceptibility gene 1 (*BRCA1*) and 2 (*BRCA2*), predispose their carriers to a higher lifetime-risk of breast and ovarian cancers [1–4]. Other cancers, which have been shown to be associated with *BRCA1/2* mutations, include male breast cancer, prostate cancer, pancreatic cancer, and melanoma [5–7]. Although the exact role of these genes in carcinogenesis has been extensively studied and is still being explored, existing data suggest that they play a key role in DNA double-strand breaks (DSBs), chromosomal stability, apoptosis and cell cycle. DSBs are most lethal form of DNA damage and had been associated with promoted aging through a variety of molecular and cellular end points, including genome structural variations, cellular senescence, and apoptosis, which may result in neurodegeneration, cancer, loss of regenerative capacity, and inflammation [8]. Mice with homozygous deletions of either *BRCA1 or BRCA2* display higher levels of cellular senescence and apoptosis and are embryonically lethal [9–10], additionally *BRCA1*+/− p53+/− mice displayed multiple premature aging phenotypes, such as osteoporosis, atrophy, kyphosis, decreased body weight, and increased tumor incidence [11].

It has been recently indicated, that female carriers of *BRCA* mutation have lower ovarian reserve, tendency to experience premature menopause, increased ovarian aging, decreased AMH level and lower ovarian response to ovarian stimulation than non-carriers [12–16]. A subsequent preclinical study indicated that the expression of key DSBs repair genes – *BRCA1, MRE11, Rad51* and *ATM*, but not *BRCA2*, decline in mouse and human oocytes. The reproductive capacity of *BRCA1*-deficient mice was impaired, the count of primordial follicles was low, and DSBs in the remaining follicles were increased with age, more than in wild type mice [17]. In contrast, Valentini et al. [18] found that *BRCA* carriers and non-carriers did not differ in the rate of chemotherapy-induced amenorrhea. Due to the unsolved controversy regarding the link between *BRCA* mutation status and accelerated ovarian aging [19], our aim was to examine serum and ovarian biomarkers for ovarian aging in young *BRCA* mutation carriers. We hypothesized that *BRCA* role in aging is not restricted to the ovary; and therefore, looked also for systemic aging biomarkers in a population of young *BRCA* carries, both females and males.

## RESULTS

### Ovarian aging in *BRCA* mutation female carriers

**Figure 1:**
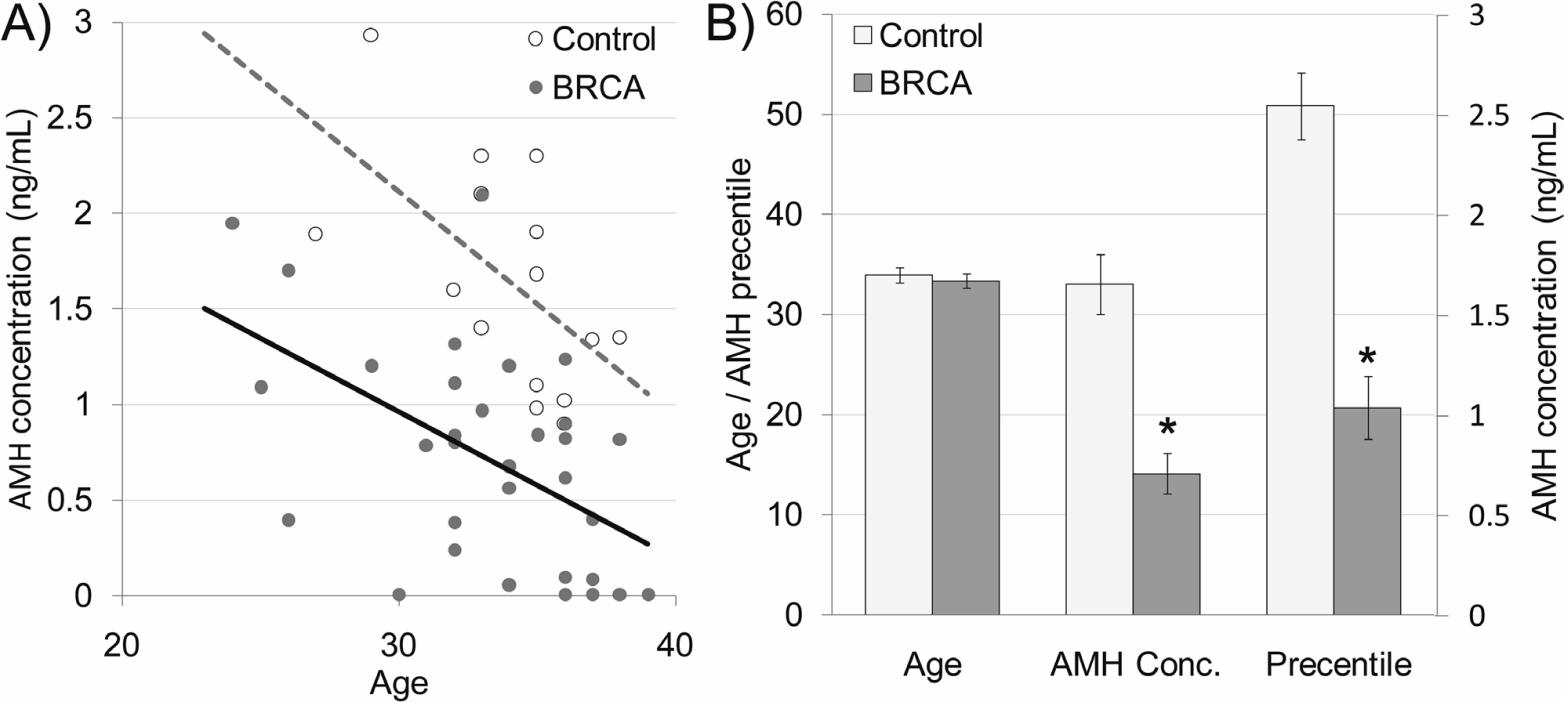
Serum anti-Mullerian hormone (AMH) in *BRCA* mutation female carriers **(A)** Scatter plot representing serum AMH levels in 15 non-carrier females (control; white dots; dashed grey regression line) and 33 *BRCA* mutation carrier females (BRCA; grey dots; continues black regression line). **(B)** Age, serum AMH concentration and AMH levels percentiles in non-carrier females (control; light gray bars) and *BRCA* mutation carriers (BRCA; dark gray bars). Each bar is mean ±SEM. ^*^ significantly different from corresponding control value (*P*<0.05).

**Figure 2:**
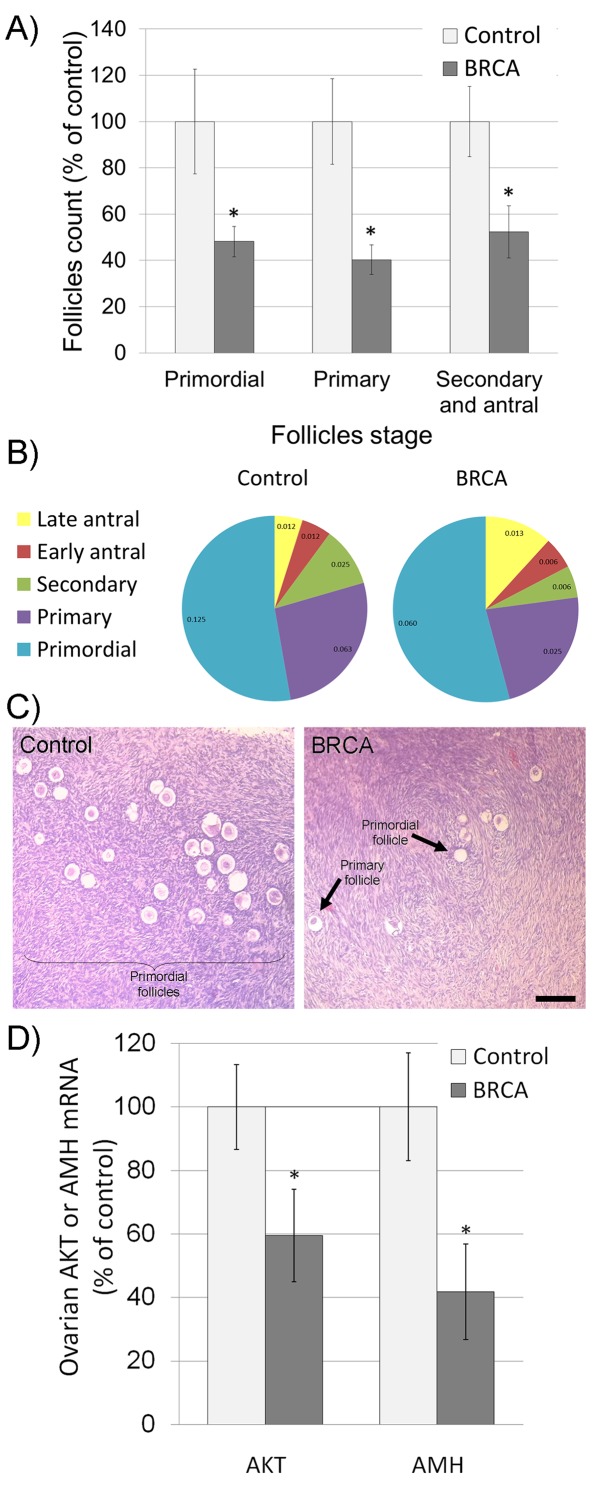
Characteristics of ovaries excised from female *BRCA* carriers **(A-B)** Excised female ovaries were fixed and stained with hematoxylin and eosin (H&E); follicles were divided into three main categories according to their size and developmental stage: primordial, primary, secondary and antral follicles. Follicles were counted according to category, in at least two transverse ovarian sections for each female [21]. The number of each type of ovarian follicle per mm2 of ovary was recorded and presented as percentage of follicular density in control non-carriers group (A; Each bar is mean ± SEM. ^*^ significantly different from corresponding control value *P*<0.05) or as percentage of total follicles in each group (B; number of follicles/mm^2^ is presented in each slice). **(C)** Representative images of H&E stained ovaries excised from non-carrier (control) and from *BRCA*-carrier patients (BRCA). Bar = 100 μm. **(D)** Levels of AKT and AMH mRNAs in ovaries of non-carriers (control; light gray bars) and *BRCA*-carriers (BRCA; dark gray bars). Bars are mean ± SEM. ^*^ significantly different from corresponding control value (*P*<0.05).

Thirty-three female *BRCA* carriers (median age 35y) and 20 male carriers (44y) were enrolled and matched to 15 non-carrier females (34y) and 16 non-carrier males (43y) as depicted in Table 1. All patients were healthy, BMI 18-22 with background of neither cardiovascular diseases nor comorbidities. Serum AMH served as an indicator of ovarian reserve in females [20] and of testicular toxicity in males [21]. AMH measurement have clinical advantage over measurements of other markers of ovarian aging, such as inhibin B, estradiol and follicle-stimulating hormone (FSH), which are all menstrual cycle dependent and mark only late ongoing process of primordial follicle pool depletion [20]. We have shown that serum AMH may serve as marker for testicular function after induced toxicity, whereas other groups demonstrated age dependent serum AMH reduction that may serve as marker for testicular aging and age-related reducing of testicular function [22–24]. Percentiles of AMH levels in females were determined according to AMH-nomograms of Tehrani et al. [25]. AMH amount was significantly lower in female *BRCA* carriers than in female non-carrier controls and continue to decrease with age (Figure 1; *P*<0.05). Interestingly, AMH level was similar in *BRCA* carriers and non-carriers (supplementary Figure 1; P>0.05). Next we examined ovarian tissues obtained from *BRCA* carriers and age-matched healthy non-carrier controls by H&E staining and morphometric analysis. *BRCA* carriers showed fewer primordial, primary, secondary and antral follicles in ovaries of than in non-carriers (Figure 2A–2C; *P*<0.05;). Moreover, the levels of AKT and AMH mRNAs as indicators of cell survival and proliferation [26] and of ovarian reserve [20], respectively, were significantly lower in ovaries of *BRCA* carriers than in non-carriers (Figure 2C; *P*<0.05).

**Table 1:** Patients’ characteristics

Participant	Participant status	Age
1	Female carrier	36
2	Female carrier	33
3	Female carrier	29
4	Female carrier	30
5	Female carrier	37
6	Female carrier	24
7	Female carrier	26
8	Female carrier	36
9	Female carrier	38
10	Female carrier	32
11	Female carrier	35
12	Female carrier	37
13	Female carrier	32
14	Female carrier	33
15	Female carrier	38
16	Female carrier	34
17	Female carrier	32
18	Female carrier	37
19	Female carrier	25
20	Female carrier	26
21	Female carrier	34
22	Female carrier	36
23	Female carrier	32
24	Female carrier	34
25	Female carrier	31
26	Female carrier	39
27	Female carrier	32
28	Female carrier	39
29	Female carrier	36
30	Female carrier	32
31	Female carrier	34
32	Female carrier	36
33	Female carrier	36
1	Female non-carrier	35
2	Female non-carrier	36
3	Female non-carrier	33
4	Female non-carrier	33
5	Female non-carrier	35
6	Female non-carrier	27
7	Female non-carrier	29
8	Female non-carrier	35
9	Female non-carrier	33
10	Female non-carrier	36
11	Female non-carrier	35
12	Female non-carrier	32
13	Female non-carrier	38
14	Female non-carrier	37
15	Female non-carrier	35
1	Male carrier	42
2	Male carrier	46
3	Male carrier	46
4	Male carrier	45
5	Male carrier	46
6	Male carrier	48
7	Male carrier	44
8	Male carrier	48
9	Male carrier	42
10	Male carrier	47
11	Male carrier	42
12	Male carrier	44
13	Male carrier	42
14	Male carrier	40
15	Male carrier	43
16	Male carrier	42
17	Male carrier	40
18	Male carrier	47
19	Male carrier	45
20	Male carrier	42
1	Male non-carrier	41
2	Male non-carrier	44
3	Male non-carrier	42
4	Male non-carrier	45
5	Male non-carrier	43
6	Male non-carrier	46
7	Male non-carrier	39
8	Male non-carrier	48
9	Male non-carrier	47
10	Male non-carrier	41
11	Male non-carrier	43
12	Male non-carrier	44
13	Male non-carrier	46
14	Male non-carrier	41
15	Male non-carrier	40
16	Male non-carrier	43

Gender, status of genetic carrying of BRCA mutation and age of 84 participants in study.

### Systematic aging in *BRCA* mutation female carriers

**Figure 3:**
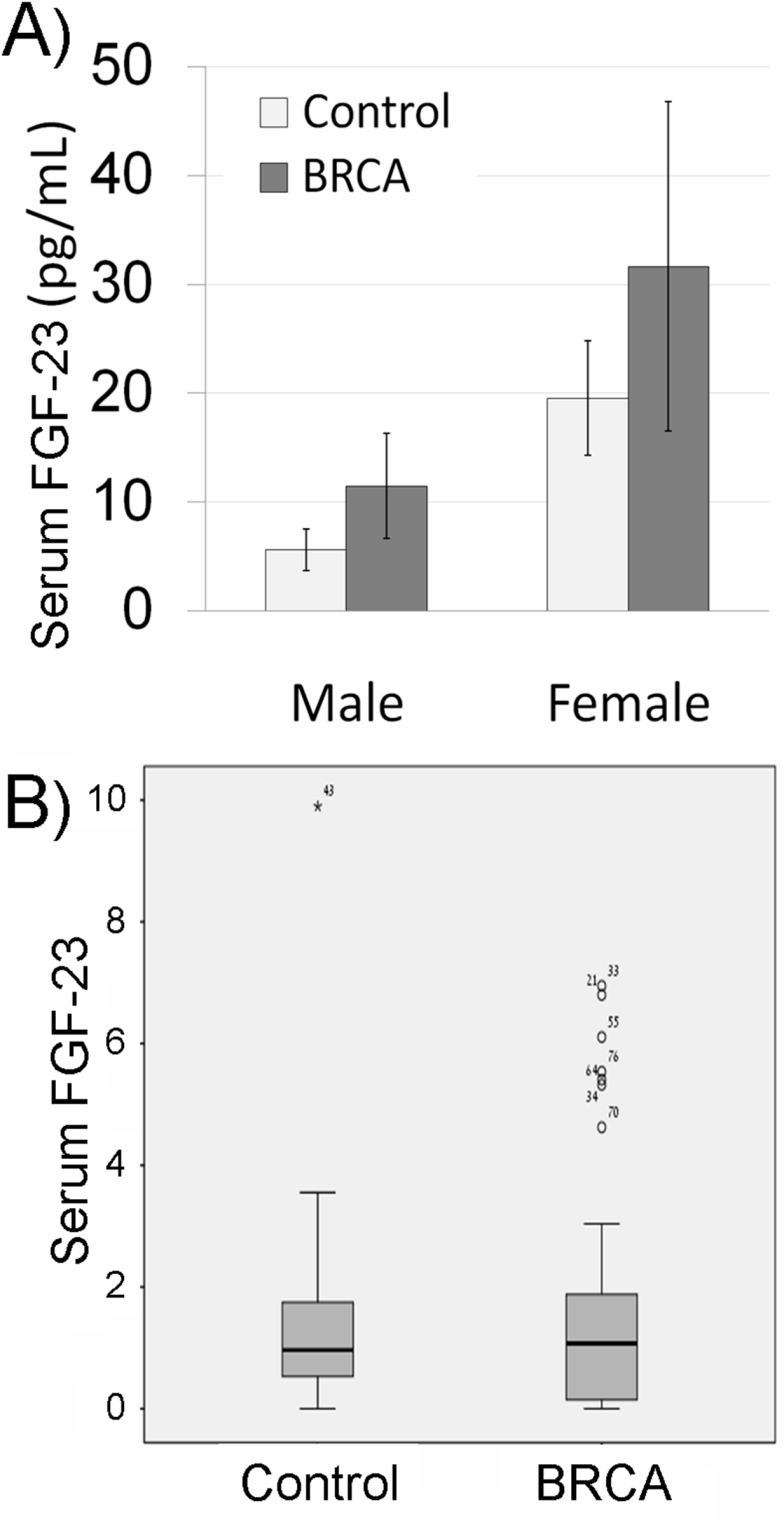
Serum fibroblast growth factor-23 (FGF-23) in *BRCA* mutation carriers **(A)** Bar chart of serum FGF-23 concentration in non-carrier females and males (control; light gray bars) and *BRCA* mutation carriers (BRCA; dark gray bars). Each bar is mean ± SEM. **(B)** Box plot of serum FGF-23 concentration in non-carriers (control; females and males) or *BRCA* mutation carriers (BRCA) normalized to the median in the cohort. Outliers are marked in asterisk or circle with the sample number.

**Figure 4:**
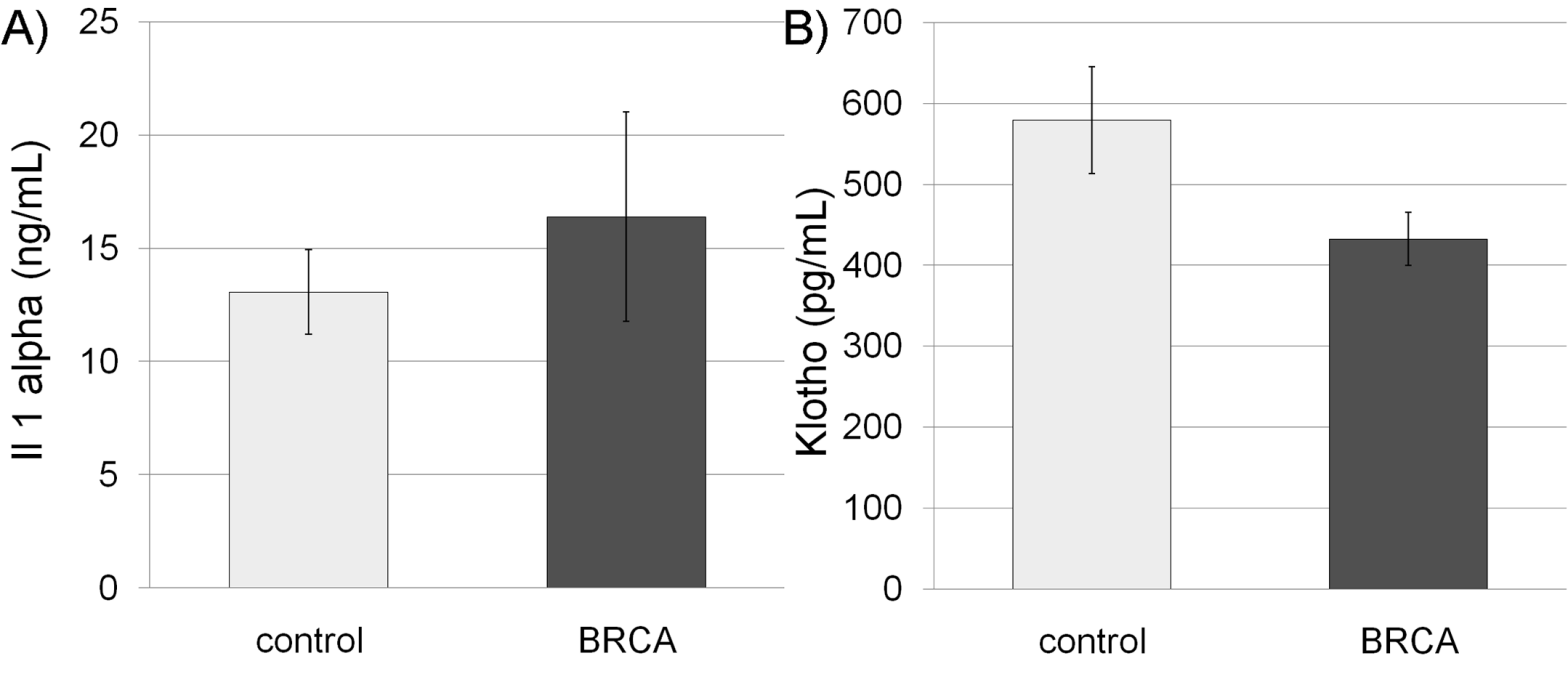
Serum interleukin 1 alpha (Il-1A) and Klotho in *BRCA* mutation carriers Bar charts of serum Il-1A **(A)** and Klotho **(B)** concentrations in non-carriers (control; light gray bars) and *BRCA* mutation carriers (BRCA; dark gray bars). Each bar is mean ± SEM.

We measured systemic aging biomarkers in *BRCA* carriers in order to examine possible involvement of BRCA in systemic aging. FGF-23 is a key molecule regulating mineral homeostasis and vascular calcification and is its concentration in serum is correlated with aging [27]. Hence, the concentration of FGF-23, known to increase with age [27], was initially examined. The median concentration of FGF-23 was higher in *BRCA* carriers of both genders than in the control group, though nearly significant (P=0.06 CI), possibly because of high variability. Because there are no established cut-off values for the level of serum FGF-23 we normalized the level of FGF in the serum to the median in our cohort. The distribution range of FGF-23 values among carriers was significantly different in *BRCA*-carriers and non-carriers. Only one non-carrier had an FGF-23 level higher than 75th percentile, whereas 8 *BRCA*-carriers depicted outlier values (p=0.04; Pearson Chi-Square test; Figure 3B). The same phenomenon was demonstrated after we performed the same analysis but stratified the subjects by gender (p=0.047; Pearson Chi-Square test). We also examined other systemic aging biomarkers such as serum Il-1A that increases with age and emerges as an important participant in aging [21] and serum Klotho that decreases with age and takes part in many systemic aging processes that also affect lifespan [28]. The level of IL-1A was higher in *BRCA*-carriers than in their corresponding controls (Figure 4A), whereas Klotho level was lower in *BRCA*-carriers than in their corresponding controls (Figure 4B). Yet, the differences in Klotho levels in carriers were not significant according to independent, two-sample two-tailed t-test for unequal sample sizes and unequal variances (P>0.05; It was found to be significant in one-tail and equal variances t-test).

## DISCUSSION

Our study sheds light on the emerging controversy regarding the role *BRCA* gene plays in the ovary, regardless of its role in the development of ovarian and other malignancies. We demonstrated that young *BRCA* carriers have lesser ovarian reserve than age-matched non-carriers. Other studies indicated that *BRCA* mutation carriers experience early menopause [15–16, 29]. A contradictory study performed by Valentini [18] determined that the risk of induced long-term amenorrhea does not seem to be greater among *BRCA* carriers than non-carriers. To note, menstruation may not be the finest indicator of ovarian reserve and may be affected by other conditions. Titus et al. [17] revealed that the function of *BRCA1* and other key DNA repair genes in oocytes declines significantly with age in mice and woman, and when *BRCA* is knocked out by gene interference in mice, it stimulates apoptosis and diminishes ovarian reserve, indicating that the efficiency of DNA DSBs repair is a key effector in oocyte demise. Our results of ovarian aging in humans, assessed by follicles count, serum AMH level, and expression of AMH and AKT, are in accordance with recent study showing primordial follicle loss in humans, possibly because of oocyte DNA damage in women with *BRCA* mutations [30]. We speculate that the general DNA damage in general and DNA double stand break in particular may also accumulate in somatic cells in the ovary and other organs and may lead to general pathologies such as systematic aging.

The link between ovarian function and aging has been well established. Menopause transition has been correlated with aging and increased risk of future cardiovascular morbidity [31–33]. AMH, which is established as a reliable marker of ovarian reserve, was found also to be reciprocally correlated with impaired lipid profile, where women in the lowest baseline age-specific AMH quartile exhibited a worsening lipid profile compared to women in the highest AMH quartile [34–35]. Nevertheless, there is still controversy regarding the magnitude of the cardiovascular risk and AMH levels in healthy adults [20, 36–37]. Another Mechanism that supports the link between diminished ovarian reserve and aging is supported by the observation that woman with shorter telomeres and reduced telomerase activity have lesser ovarian reserve [38]. Moreover, Meta-analysis of 13 loci associated with age at menopause revealed that these gene loci were related to DNA repair, an important contributor to somatic aging [39]. It has been recently shown that *BRCA* gene products have a role in telomere length homeostasis in healthy *BRCA* carriers [40]. We presumed that ovarian aging might reflect a broader systemic phenomenon evaluated whether *BRCA* carriers and non-carriers differ in their expression pattern of aging-related factors, as FGF-23, IL-1A and Klotho. Assessment of systemic aging is considered highly challenging. The range of laboratory values and wide variability among subjects is a known Achilles’ heel. While FGF, Klotho and IL1 may serve as standard surrogate biomarkers for systemic aging [21, 27–28] other well-established markers may also serve for such purpose. Since ovarian aging may contribute to systemic somatic aging, we also included male *BRCA* carriers in our analysis to exclude ovarian effect. We observed a remarkable trend of increased median FGF-23 levels in the serum of *BRCA* carriers (of both genders), compared to age-matched non-carriers. Klotho levels in both genders were lower in the serum of *BRCA* carriers than in non-carriers, though they did not reach statistical significance. The levels of IL-1A in the serum depicted a reciprocal pattern: higher in *BRCA* carriers, though due to high variability it also did not reach statistical significance. Limited by small sample size, the patterns yielded in our study were very obvious, yet not clearly significant, hampered by high variability. Aging is also characterized by increased cardiovascular risk, due to endothelial dysfunction via increased generation of reactive oxygen species (ROS), thus elevating oxidative stress that in turn promotes DNA damage and endothelial cell apoptosis [41]. It had been recently demonstrated that *BRCA* mutation carriers had increased risk of chemotherapy-induced cardiotoxicity in both mouse and human [41, 42]. The pre-clinical data obtained in mice highlight an unrecognized role of *BRCA* as a gatekeeper of inflammation-induced endothelial cell function and a target to limit atherosclerosis [43–46]. *BRCA1* is expressed in endothelial cells in mice and overexpression of *BRCA1* strongly attenuated the production of ROS and AKT. Moreover, a Canadian study estimated that in the absence of melanoma, breast, ovarian and pancreatic cancers, the life expectancy of female and male *BRCA* carriers was 6.8 and 3.7 years lower than that of non-carriers, respectively [47]. An overall test of association for men and women together showed a statistically significant association between *BRCA1/2* mutations and increased non-cancer mortality, complementing our data.

In conclusion, our results lend credence to a newly paradigm, that suggests a potential link between *BRCA* mutation, accelerated gonadal aging and systemic aging-related pathophysiology. Because DNA damage and repair plays an essential role in carcinogenesis, it also represents a common contributor for aging-related conditions characterized by increased cellular and oxidative stress manifested by DNA damage. Thus, *BRCA* carriers may be predisposed to enhanced aging, potential endothelial dysfunction and related vascular morbidity. Future studies should elucidate this hypothesis and appraise the prevalence of non-cancer morbidities in *BRCA* carriers, as these data may carry a significant implication for preventive and early detection measures for this population.

## MATERIALS AND METHODS

### Experimental design in patients

Study participants were healthy *BRCA* carriers; 33 females, 40 years or younger, before prophylactic oophorectomy (PO) and 20 males, 50 years or younger. Control cohorts were consisted of age-matched healthy non-carriers of each gender (15 females and 16 males). The protocol was approved by Rabin Medical Center (RMC) institutional review board (RMC-6103) and all patients signed a relevant informed consent form. Blood samples were drawn from both genders for hormones and cytokines measurements. Ovarian tissue obtained from *BRCA* carriers who underwent PO, and from age-matched healthy non-carriers, who underwent partial oophorectomy due to benign conditions, were processed for histological and gene expression analyses.

### Measurements of serum hormones and cytokines

Blood samples were centrifuged (6000 rpm, 10 min, 4°C) and serum was stored in aliquots at −20°C until measurements. Anti-Mullerian hormone (AMH; Beckman Coulter, Chaska, MN, USA), fibroblast growth factor-23 (FGF-23; Immutopics, San Clemente, CA, USA), interleukin 1 alpha (Il-1A; R&D Systems, Minneapolis, MN, USA) and Klotho (Immuno-Biological Laboratories, Minneapolis, MN, USA) were measured by enzyme-linked immunosorbent assay (ELISA) according to the manufacturer’s instructions. Calibrators for a standard-curve as well as low and high controls were added in duplicates to each ELISA plate. Samples were measured by the SpectraMax 190 microplate-reader (Molecular Devices, Sunnyvale, CA, USA).

### Histological staining and morphometric analysis of ovaries

Ovaries were fixed in 4%; paraformaldehyde (Merck, Darmstadt, Germany), embedded in paraffin, sectioned (8 μm) and mounted on Superfrost/Plus slides (Daigger and Co., Wheeling, IL, USA). Several transverse sections from each ovary were stained with hematoxylin and eosin (H&E)[48]. Bright-field images were recorded by a digital-camera (Canon pc1089 CCD, Tokyo, Japan) connected to an Axiovert 200M inverted microscope (Carl Zeiss MicroImaging; Oberkochen, Germany) equipped with an Apochromat 20X objective. Primordial, primary, secondary and antral follicles amount was counted as previously described [21] in at least two transverse ovarian sections for each female. Assessments of the ovaries were performed by two independent evaluators, clinical pathologist and clinical embryologist and histology expert (level of agreement was higher than 50%). The number of each type of ovarian follicle per mm^2^ of ovary was recorded and presented as percentage of follicular density in control group. The total volume of the ovaries was not assessed.

### Ovarian gene expression analysis by qPCR

Purification of total RNA from formalin fixed, paraffin-embedded microdissected ovarian tissue was made by RNeasy-FFPE kit according to the manufacturer’s instructions (QIAGEN GmbH, Hilden, Germany). Ovarian mRNA was quantified [21]; first-strand cDNA was created by RT (Applied biosystems, Foster City, California) in 35 cycles with 0.4 μM gene-specific primers using ready-mix mixture (Sigma). mRNA amount were assessed by SYBR green reagent (SYBR Green PCR Master Mix, ABI, Carlsbad, CA, USA) on an ABI Prism 7900 Sequence PCR machine. In each run, 20 ng of cDNA per reaction were used as an amplification template. The house-keeping gene selected for the qPCR calibration was hypoxanthine-guanine phosphoribosyltransferase (HPRT1). The primers used were as follows: human HPRT1 forward 5’ TGA CAC TGG CAA AAC AAT GCA 3’; human HPRT1 reverse 5’ GGT CCT TTT CAC CAG CAA GCT 3’; human Protein kinase B (AKT) forward 5’ ACG TGG CTA TTG TGA AGG AG 3’; human AKT reverse 5’ CAT TCT TGA GGA GGA AGT AGC G 3’; human AMH forward 5’ CAG TTG CTA GTC CTA CAT CTG GCT GA 3’; human AMH reverse 5’ GGA AGT CCA CGG TTA GCA CCA AAT 3’. Data was recorded and analyzed by StepOne 2.1 software (Applied biosystems).

### Statistical analysis

Data of serum AMH, Il-1A and Klotho levels, follicular count and mRNA levels was evaluated by independent, two-sample two-tailed t-test for unequal sample sizes and unequal variances with significance of *P* < 0.05. A correlated one-way ANOVA statistical analysis showed similar results. Data of detected or undetected serum FGF-23 levels was also evaluated by Pearson Chi-square test.

### Statement of significance

*BRCA* carriers may be potentially predisposed to enhanced aging.

## SUPPLEMENTARY MATERIALS FIGURES


