# Supplementary material for: Premature ovarian aging in BRCA carriers: a prototype of systemic precocious aging?
Source: Oncotarget. 2018 Mar 23;9(22):15931–41. doi: 10.18632/oncotarget.24638 (PMC5882308; doi:10.18632/oncotarget.24638)
Supplement: Supplementary file 1 [file oncotarget-09-15931-s001.pdf]

Premature ovarian aging in *BRCA* carriers: a prototype of systemic precocious aging?

SUPPLEMENTARY MATERIALS

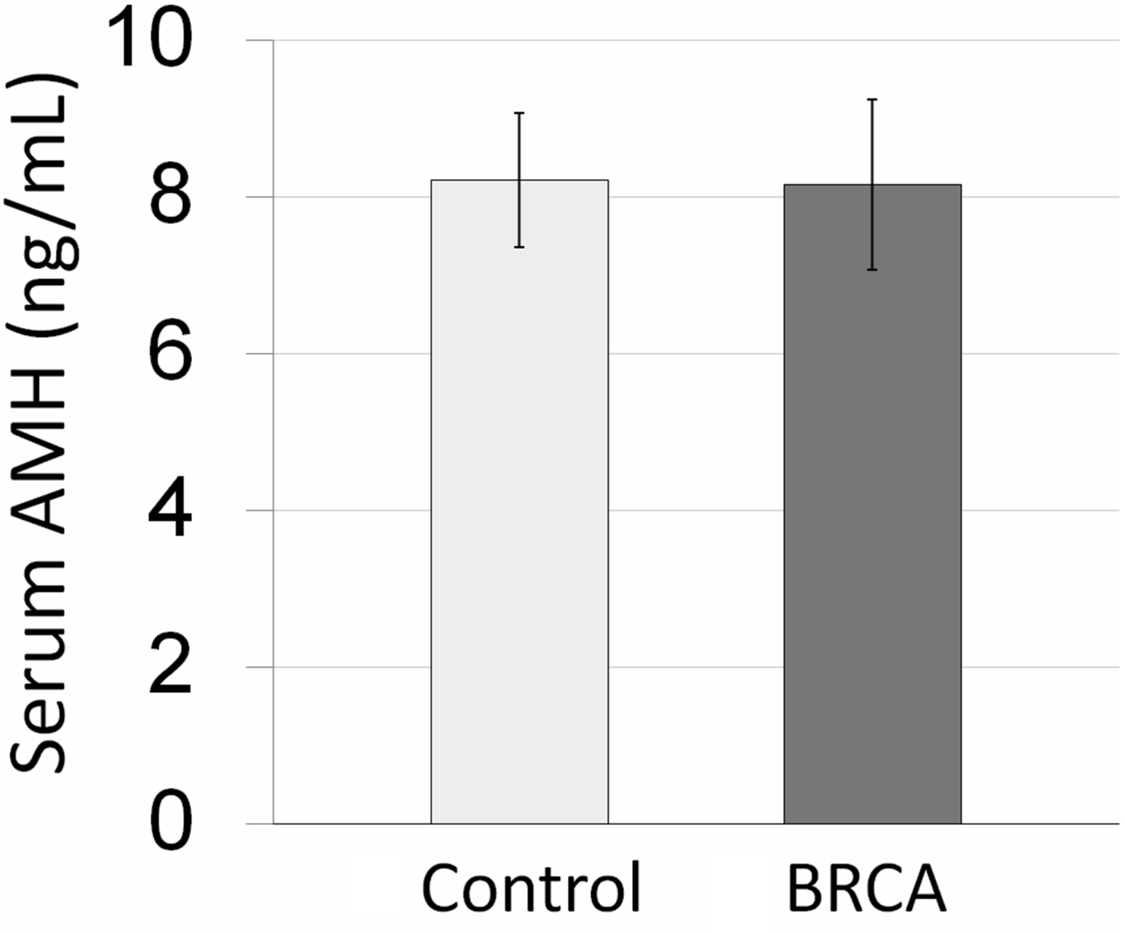

Supplementary Figure 1: The level of anti-Mullerian hormone (AMH) in the serum of male *BRCA* mutation carriers. AMH concentration in the serum of male non-carriers (control; light gray bars) and *BRCA* mutation carriers (BRCA; dark gray bars). Each bar is mean ± SEM.
